# Listen to yourself! Prioritization of self‐associated and own voice cues

**DOI:** 10.1111/bjop.12741

**Published:** 2024-10-03

**Authors:** Neil W. Kirk, Sheila J. Cunningham

**Affiliations:** ^1^ Division of Sociological and Psychological Sciences Abertay University Dundee UK

**Keywords:** own voice, self, self‐association, self‐prioritization effect, vocal cues

## Abstract

Self‐cues such as one's own name or face attract attention, reflecting a bias for stimuli connected to self to be prioritized in cognition. Recent evidence suggests that even external voices can elicit this self‐prioritization effect; in a voice‐label matching task, external voices assigned to the Self‐identity label ‘you’ elicited faster responses than those assigned to ‘friend’ or ‘stranger’ (Payne et al., Br. J. Psychology, 112, 585‐610). However, it is not clear whether external voices assigned to Self are prioritized over participants' own voices. We explore this issue in two experiments. In Exp 1 (*N* = 35), a voice‐label matching task comprising three external voices confirmed that reaction time and accuracy are improved when an external voice cue is assigned to Self rather than Friend or Stranger. In Exp 2 (*N* = 90), one of the voice cues was replaced with a recording of the participant's own voice. Reaction time and accuracy showed a consistent advantage for the participant's own‐voice, even when it was assigned to the ‘friend’ or ‘stranger’ identity. These findings show that external voices can elicit self‐prioritization effects if associated with Self, but they are not prioritized above individuals' own voices. This has implications for external voice production technology, suggesting own‐voice imitation may be beneficial.

Our own voice is a powerful marker of our identity, such that those who have lost or never had the ability to speak can face significant challenges in self‐representation and social interaction. Assistive communication devices offer these individuals the ability to produce speech, but often in a manner that sounds impersonal and artificial. For example, a recent report highlights the case of a young man (DC) with cerebral palsy, who struggled with the robotic voice provided by his device (Jones & Bottomley, 2024). After launching an appeal for volunteers with the regional accent spoken by his family, DC found a voice that allows him to ‘thrive now he finally has his own identity’. While this case highlights the practical benefits of adopting a new externally generated voice, it also raises important theoretical questions about vocal self‐biases. Can externally produced voices truly become part of one's self‐representation and do they elicit similar or stronger cognitive biases compared to one's own voice?

While there are well‐established cognitive biases to self‐cues such as attention capture by one's own name and own face, little empirical attention has been paid to the processing of one's own voice (Xu et al., 2013). This is somewhat surprising given the unique nature of individual voices, which serve as personal identifiers and are perceived as conveying key characteristics (e.g. gender, social class, personality traits; see Lavan, 2023, McAleer et al., 2014). Our own voice is also a highly familiar verbal cue, such that people report uncomfortable dissonance when exposed to the change in sound associated with hearing an external recording of one's own voice compared to natural self‐vocalization heard internally (Holzman & Rousey, 1966; Kimura & Yotsumoto, 2018). Despite this acoustic variation, people are able to reliably distinguish recordings of their own voice from external voices (Gur & Sackeim, 1979; Xu et al., 2013), and there is evidence of overlap in the neural responses typically evoked by own‐voice cues and visual self‐cues such as own‐face images, when contrasted with the response to cues associated with other people (Conde et al., 2019; Rosa et al., 2008; Uddin et al., 2007).

Self‐related stimuli are associated with prioritization in cognition, with self‐cues eliciting faster perception and enhanced attention, and items encoded with reference to self being better remembered than those encoded in other contexts (see Humphreys & Siu, 2016). These effects are often investigated using familiar cues associated with self across time (e.g. own names), which may be underpinned by conditioned physiological reactions such as hearing one's own name being associated early in development with an expectation of response (see Kaida & Abe, 2018). However, stimuli temporarily associated with self through ownership or task‐specific instructions are also prioritized in cognition, eliciting attentional and memorial bias (Cunningham et al., 2008; Sui et al., 2012). For example, in Sui et al.'s (2012) shape‐matching task, participants learn three arbitrary associations between shapes and referents including themselves (e.g. ‘you are the triangle, friend is the square, stranger is the circle’). They are then presented with shape‐label pairings on‐screen (e.g. triangle + ‘You’) and asked whether the pairing is a match or mismatch. Participants reliably respond more quickly and accurately to the shapes assigned to the ‘self’ identity label than those assigned to the other referent identities, a bias known as the self‐prioritization effect (SPE; Sui et al., 2012).

Extending SPE research to vocal cues, recent work has explored the extent to which the SPE could be elicited by externally generated voices assigned to self and other identities. Payne et al. (2021) adapted the shape‐matching task by replacing the shapes with recorded vocalizations of the word ‘hello’ provided by three individuals unknown to participants. Thus, participants learned arbitrary associations between three distinct voice cues and the labels ‘you’, ‘friend’ and ‘stranger’. Across three experiments, they were presented with voice cue and label pairings and asked to determine whether each pairing was a match or mismatch. Response time and accuracy data revealed a reliable bias for the voice assigned to the self identity, an auditory SPE, demonstrating that voices did not have to be self‐generated to elicit this effect.

Evidence for an auditory SPE suggests that externally generated voices can become cognitively associated with self, such that they elicit self‐biases in attention. An important question is whether the association with self is stronger when it shares characteristics with self, such as gender, accent or vocal patterns. In the age of AI‐generated voices and personalization of digital self‐cues, it is becoming increasingly possible to match externally produced voices with individual vocal characteristics, such that synthesized voices are more recognizably one's own (see Snyder et al., 2023). It is possible that using an external voice that is more closely aligned with self could increase the level of SPE elicited by such devices. Interestingly, Payne et al. (2021, Exp 2) found that same‐gender voices were not effective at enhancing the SPE, but this may be because gender matching was insufficient to produce auditory resemblance, as well as gender being task‐irrelevant and therefore unlikely to be an active social identity for participants (Spears, 2011). The question remains then, whether the SPE can be enhanced when the voice cues are more closely aligned with self.

Here, we report two experiments that explore this question using natural voices. Exp 1 sought to replicate Payne et al.'s (Exp 1) findings that voices belonging to other referents can elicit an SPE even if they do not match one's own voice. As the published literature on the SPE for voices is limited, close replication is needed to determine the validity of the voice‐matching paradigm (see Clarke, et al., 2023). Extending the paradigm, in Exp 2, a novel manipulation compared the SPE elicited by recordings of participants' own voices with those temporarily assigned to Self. Recent research suggests that temporary task goals can override the prioritization of familiar self‐cues such as own‐face images (Cunningham et al., 2022; see also Golubickis & Macrae, 2023), suggesting that a temporary self‐association may be a stronger cue in Payne et al.'s task than an own‐voice cue assigned to other referents. However, additional links with self may boost prioritization (see Cunningham et al., 2011; Payne et al., 2021 Exp 3), leading to a prediction that own‐voices should elicit the strongest effects when assigned to Self. The current study therefore aimed to establish the extent to which external voices assigned to Self and participants' own‐voices elicit self‐bias both independently and in combination.

All experiments were conducted in the ethos of open and reproducible science. The experimental design, methods and analysis plans were pre‐registered prior to data collection on the Open Science Framework (OSF). All data, analysis scripts and Appendix S1 are publicly available in a dedicated OSF repository (https://osf.io/hr96d/?view_only=090108f5331c4469b3ee5b78d99a50ae). Study materials are available as Open Materials on the Gorilla Experiment Builder platform (https://app.gorilla.sc/openmaterials/740669). The experiments were approved by Abertay University's Faculty Research Ethics Committee (EMS6415 and EMS6444).

## EXPERIMENT 1

Exp 1 was a close replication of Payne et al.'s (2021) Exp 1, in which three unknown externally generated natural voices were assigned to self, friend and stranger identity labels in a voice‐matching task, using Scottish rather than English‐accented speakers as a first step towards increasing the dialectal diversity and generalizability of these findings (see: Kirk, 2023).

Following Payne et al., the experimental hypothesis was that there would be an auditory SPE with participants having faster reaction times and greater recognition accuracy to voice cues as assigned to the identity label ‘You’, compared to those assigned to either ‘Friend’ or ‘Stranger’. For consistency, the externally generated voices were matched to participants on key social categories, so the experiment was conducted with male Scottish participants, using stimuli from male speakers with Scottish accents.

### Method

#### Participants and design

In line with our pre‐registered target, we recruited 35 male participants (mean age: 30.8 years, *SD* = 7.0, range = 18–40), based on the sample size and power analysis reported by Payne et al.'s (2021) Exp 1. Participants were recruited through Prolific (www.prolific.ac) with eligibility based on the following self‐reported criteria: Age: 18–40 years, Sex: Male, Gender Identity: Male, Nationality: Scottish, UK Area of Birth: Scotland, Current UK Area of Residence: Scotland, Hearing Difficulties: No, Prolific Approval Rate: 99–100, English Speaking Monolingual: ‘I only know English’. We targeted monolingual participants to reduce any potential effects that might be associated with bilingual language processing (e.g. inhibitory language control, Declerck & Koch, 2023). The experiment was presented using the Gorilla Experiment Builder platform (Anwyl‐Irvine et al., 2019) and participants had to pass a headphone check to proceed with the experimental trials. No participants were excluded on this or any other basis.

The experiment had a repeated measures design with all participants completing trials across all Trial Type (Match, Mismatch) and Voice identity (Self, Friend, Stranger) conditions.

#### Materials

Materials were adapted from Payne et al.'s (2021, Exp 1) experimental framework (available at https://app.gorilla.sc/openmaterials/45935). Three male speakers were recruited to provide voice samples consisting of two auditory tokens of ‘hello’, saved as wav files with 16‐bit sample width and a sampling rate of 48 kHz. Our version is available as Open Materials from: https://app.gorilla.sc/openmaterials/740669, and an online demonstration of the main task can be accessed from: https://research.sc/participant/login/dynamic/806298BD‐7C68‐4694‐A085‐CA593AF5CF54.

#### Procedure

Participants provided informed consent then completed a headphone check based on Woods et al. (2017) that required them to identify which of three tones is the quietest in a task designed to be easy over headphones but difficult over loudspeakers. Participants had to correctly identify the tone in five out of six trials to pass and continue with the experiment.

Participants were then branched into one of six different versions of the main task, which accounted for all possible combinations of the three external speakers' voices in each of the Voice identity conditions (‘you’, ‘friend’ or ‘stranger’). For each individual participant, the assignment of voices to the voice identity conditions remained the same throughout the task. Following Payne et al. (2021, Exp 1), the task involved a familiarization phase followed by a test phase.

##### Familiarization phase

Across 12 trials, participants were passively exposed to the three external voices with their assigned Voice identity (‘you’, ‘friend’ or ‘stranger’), presented in a random sequence an equal number of times. Participants were not instructed to imagine a specific referent in each condition, being told simply: ‘In this experiment you will hear three different voices. One voice belongs to YOU. One voice belongs to your FRIEND. One voice belongs to a STRANGER.’. In each familiarization phase trial, a 500 ms fixation cross appeared centrally on a white background before the Voice identity label (‘YOU’, ‘FRIEND’ or ‘STRANGER’) was presented for 3000 ms in black uppercase letters. After 500 ms, the 500‐600 ms auditory sample (‘hello’) from the associated voice was played. Including the on‐screen instructions, the familiarization phase took roughly 1 min to complete and led directly into the test phase.

##### Test phase

Each trial began with a 500 ms central fixation cross followed by a 500‐600 ms audio sample (one of the speakers saying ‘hello’). Immediately after the offset of this audio file, a label (‘you’, ‘friend’ or ‘stranger’) was displayed centrally and participants were asked to indicate whether the speaker's voice matched the label by pressing the left arrow key for ‘MATCH’ and the right arrow key for ‘MISMATCH’. Immediately after each response, on‐screen feedback was displayed for 500 ms: a green checkmark (correct response), a red cross (incorrect response) or the phrase ‘TOO SLOW’ (responses over 1500 ms). The next trial began after the 500 ms feedback period.

After the 12 practice trials, participants completed three blocks of 72 experimental trials. These were identical to the structure of practice trials, other than the response label reminders no longer being presented on screen. The test phase began with 12 practice trials in which the response labels ‘MATCH’ and ‘MISMATCH’ were presented on the left‐ and right‐hand side of the screen as a reminder, followed by three blocks of 72 experimental trials with no on‐screen response label reminders. Participants completed an equal number of match vs. mismatch trials accounting for all combinations of speaker voice and identity label, presented in a randomized order. Percentage accuracy feedback was provided at the end of each block. After all three blocks were completed, participants completed a short demographic questionnaire and then were debriefed about the nature of the study.

#### Analysis

We used RStudio (RStudio Team, 2023) and the R‐packages kableExtra (Zhu, 2023), tidyverse (Wickham et al., 2019), and ggplot2 (Wickham, 2016) and RColorBrewer (Neuwirth, 2022) for data preparation and visualization. For analysis, we used the lme4 (Bates et al., 2015), broom.mixed (Robinson & Hayes, 2023) and afex (Singmann et al., 2022) packages to conduct and report linear mixed effects models.

For consistency with Payne et al. (2021), we employed the likelihood ratio test (LRT) method for comparing model fits and reporting main effects and interactions. For consistency and comparability with previous findings, our pre‐registered analyses used the same model structures reported by Payne et al. (2021, 2024), which included random effects of participants. For reaction time data, these models were also run using the Satterthwaite method to report effect sizes for main effects and interactions, using the effectsize package (Ben‐Shachar et al., 2023) as effect sizes cannot be computed from the LRT method. Finally, for both reaction time and accuracy data, we conducted post‐hoc tests and reported Cohen's *D* effect sizes using the emmeans (Lenth, 2022) and effsize (Torchiano, 2023) packages.

### Results and discussion

Reaction time and accuracy analyses are presented here with full model specifications and outputs available in the Appendix S1. We also pre‐registered analysis of sensitivity data (*D′*) but as this reflected the patterns found in reaction time and accuracy data, for conciseness these results are presented as Appendix S1. The mean reaction time and proportionate accuracy across voice identities in match and mismatch trials are summarized in Table 1, which shows each combination of trial type (MATCH, MISMATCH) and Voice identity (Self, Friend, Stranger).

**TABLE 1:** Mean reaction time (ms) and proportionate accuracy, with 95% confidence intervals in parentheses.

Trial type	Voice identity	Reaction time	Accuracy
MATCH	Self	579.6 [567.6, 591.6]	0.95 [0.94, 0.96]
	Friend	636.9 [623.4, 650.4]	0.92 [0.90, 0.94]
	Stranger	640.6 [627.1, 654.1]	0.90 [0.88, 0.92]
MISMATCH	Self	682.4 [669.1, 695.8]	0.91 [0.89, 0.93]
	Friend	689.1 [676.2, 702.1]	0.88 [0.86, 0.90]
	Stranger	685.1 [672.2, 697.9]	0.88 [0.86, 0.90]

#### Reaction time

RTs were measured from the onset of the voice label until response submission. In line with convention from previous research (e.g. Payne et al., 2021, 2024) and our pre‐registered analysis plan, we removed incorrect responses (9.3% of trials) and outliers in which reaction times were under 200 ms or over 1500 ms (a further 0.6% of trials). The remaining trials were submitted to the following linear mixed effect model:

$$\mathrm{rt}.\sim 1+\text{voice}\_\text{identity}\times \text{trialtype}+\left(1\;|\;\text{Participant}\;\mathrm{ID}\right).$$

The full model output is available in Table S1. The model revealed a main effect of Trial Type (*χ* ^2^ (1) = 188.95, *p* < .001, *η* ^2^ = 0.03), demonstrating that MATCH (mean = 618.5, 95% CI [610.9, 626.0]) trials had quicker responses than MISMATCH trials (mean = 685.5, 95% CI [678.0, 693.0]). There was also a main effect of Voice identity (*χ* ^2^ (2) = 43.57, p < .001, *η* ^2^ = 0.01) with responses to voices assigned to the Self identity being faster than responses to both Friend (*p* < .001, *d* = −0.30, 95% CI [−0.54, −0.06]) and Stranger voices (*p* < .001, *d* = −0.31, 95% CI [−0.64, 0.02]) and no difference between Friend and Stranger voices (*p* = .911, *d* = −0.01, 95% CI [−0.28, 0.26]).

**FIGURE 1:**
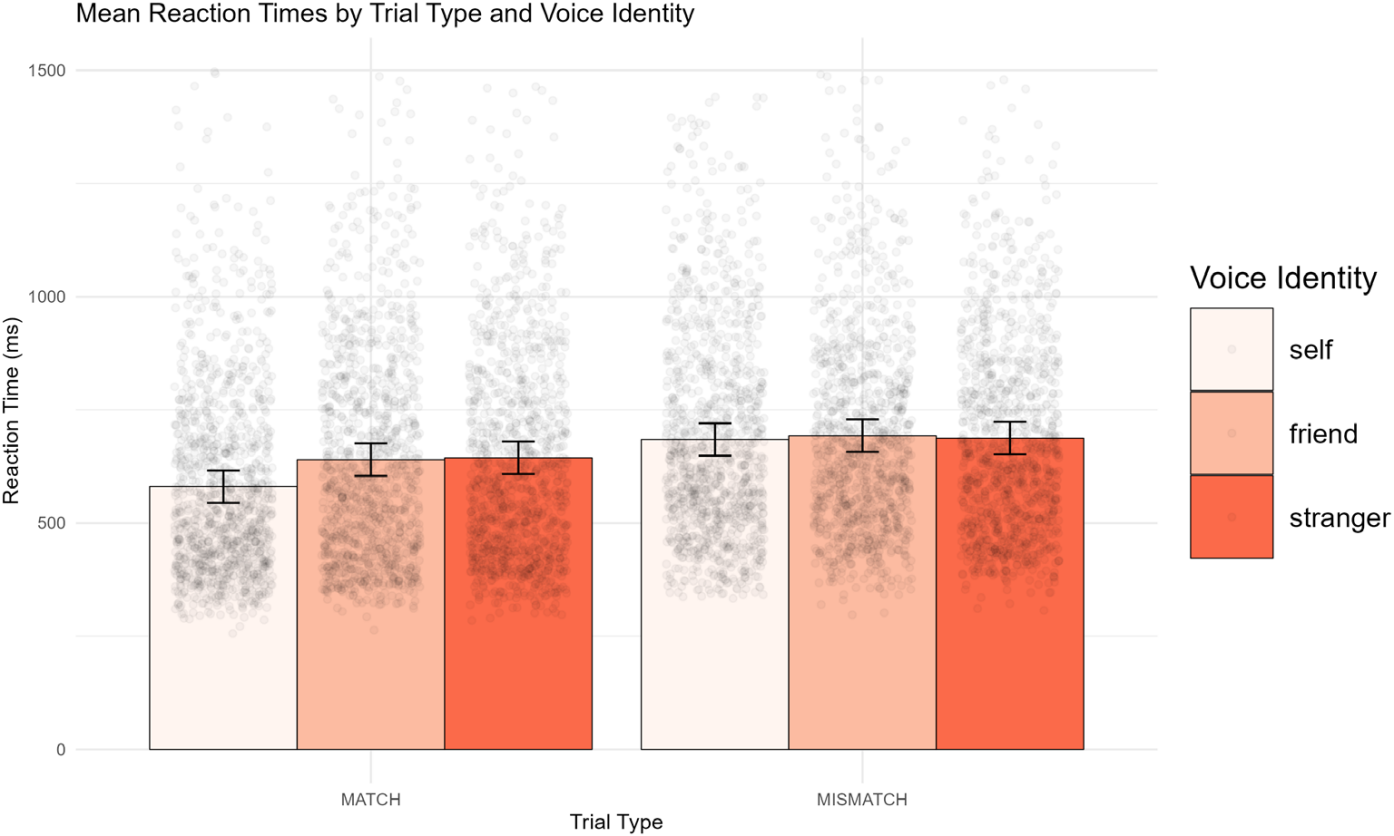
Mean RTs across trial types and voice identities. Error bars indicate 95% confidence intervals and raw data points are overlaid.

There was a significant Voice identity × Trial Type interaction (*χ* ^2^ (2) = 30.74, *p* < .001, *η* ^2^ = 0.005). Follow‐up tests of voice identity (with a Bonferroni‐corrected alpha threshold: *p* < .008) confirmed significant differences between voice identities in MATCH but not MISMATCH trials (see Figure 1). In MATCH trials, participants responded significantly faster to voices assigned to the Self identity than to Friend (*p* < .001, d = −0.46, 95% CI [−0.71, −0.22]) or Stranger voices (*p* < .001, *d* = −0.54, 95% CI [−0.90, −0.18]), with no difference between Friend and Stranger voices (*p* = .614, *d* = −0.05, 95% CI [−0.34, 0.24]). In MISMATCH trials, reaction times did not significantly differ between any of the voice identity conditions (all *p*s > .313, with Cohen's values ranging from −0.09 to 0.03). Data from previous self‐prioritization tasks in the visual domain also show that Match trials are more sensitive to differences between experimental conditions than Mismatch trials (e.g. Sui et al., 2012), so this null effect is not unusual. Overall, these patterns replicate Payne et al.'s (2021) RT findings, with Match trial responses showing an SPE for externally generated voices.

#### Accuracy

Accuracy data were submitted to the following general linear mixed effect (see Table S2 for full model output):

$$\text{Correct}\sim 1+\text{voice}\_{\text{identity}}^{*}\;\text{trialtype}+\left(1\;|\;\text{Participant}\;\mathrm{ID}\right).$$

The model revealed a main effect of Trial Type (*χ* ^2^ (1) = 27.32, *p* < .001) indicative of higher accuracy in the MATCH than MISMATCH trials. There was also a main effect of Voice identity (*χ* ^2^ (2) = 30.00, *p* < .001), with post‐hoc tests (Bonferroni‐adjusted) revealing higher accuracy for voices assigned to the Self identity than both Friend voices (*p* < .001, *d* = 0.42, 95% CI [0.14, 0.70]) and Stranger voices (*p* < .001, *d* = 0.51, 95% CI [0.16, 0.86]), while Friend and Stranger voices did not differ (*p* = .217, *d* = 0.10, 95% CI = [−0.21, 0.40]). The Trial Type × Voice identity interaction was not significant (*χ* ^2^ (2) = 2.74, *p* = .254). This suggests that unlike RT, the effect of Voice identity on accuracy was not dependent on the type of trial, although differences in accuracy across condition may have been masked by ceiling effects. As with the RT data, the accuracy findings in the current study replicate those reported by Payne et al. (2021).

Overall, Exp 1 findings show that the SPE is robust in the voice‐matching task, emerging in Match trials in the RT data and across trial types in Accuracy. While replicating Payne et al.'s (2021) SPE findings with new voices is important, a key purpose of the current research was to investigate the extent to which voice cues assigned to the self identity are prioritized in relation to participant's own voices. A second experiment was therefore conducted to contrast responses to own‐voice cues and external voice cues assigned to the self‐identity.

## EXPERIMENT 2

Experiment 2 was designed to investigate whether the long‐term association of physical own‐voice cues or cues temporarily assigned to self are prioritized. Previous research suggests that self‐prioritization effects are dynamic, capable of being attenuated by top‐down attentional goals (Golubickis & Macrae, 2023; Humphreys & Siu, 2016). For example, using a dot‐probe task, Cunningham et al. (2022) showed that participants had an attention bias towards images of their own face relative to other face images, but when participants were instructed to monitor for the presence of another ‘goal’ image, the self‐bias was attenuated as the goal image took attentional priority. Interestingly, it was found that when the goal image was of the participant's own‐face, the attentional bias was significantly boosted relative to other goal images. This suggests that self‐biases can be additive: a temporary goal combined with a self‐associated cue evoked the greatest effect on attention. Applying the same logic to the voice‐matching task would suggest that when a participant's own voice is assigned to the self identity, this should elicit the fastest and most accurate responses. To test this proposal, in Exp 2 a mixed design was applied, with a between‐subjects ‘Own‐voice assignment’ manipulation and the repeated measures Voice identity and Trial type factors. In the ‘Own‐voice assignment’ factor, some participants would have their own voice assigned to the self‐identity, whereas others would have an external voice assigned to the self‐identity, but their own voice assigned to the friend or stranger identities.

### Method

#### Participants and design

In total, 126 participants were recruited to provide voice recordings based on our pre‐registered power analysis^1^ (see: https://osf.io/hr96d/?view_only=090108f5331c4469b3ee5b78d99a50ae). Participants were recruited via Prolific using the following criteria: Age 18–40; Sex: Male; Gender Identity: Male; Nationality: English; UK Area of Birth: East of England, South East England; Current UK Area of Residence: East of England, London, England, South East England, South West England; English Speaking Monolingual: ‘I only know English’; Hearing Difficulties: No; Prolific Approval Rate: 99–100. This task lasted approximately 5 min, and participants were paid £0.90 GBP.

All 126 of the participants who provided a voice recording were invited to the experimental testing phase, comprising a voice‐matching task incorporating their own voice recordings. Thirty‐six of these participants were not included in the final sample: three did not provide sufficiently clear voice recordings, 10 did not pass the headphone check, and 21 did not respond within the data collection period. An additional two participants were removed for having chance‐level accuracy and unusually fast response times, indicative of non‐compliance with the task or bot‐like behaviour. The final sample therefore comprised 90 participants (mean age: 31.3 years), who were each paid £3.00 GBP for completing the experimental task.

The experiment had a mixed design with one between‐subject factor of Own‐voice assignment (SELF, FRIEND, STRANGER) and two repeated measures factors of Voice identity (Self, Friend, Stranger) and Trial Type (MATCH, MISMATCH).

#### Materials

We used recordings from two English‐accented male speakers (labelled M5 and M10) from Payne et al.'s (2021) Experiment 1, which were available as open materials on the Gorilla Experiment Builder platform (https://app.gorilla.sc/openmaterials/45935). We also adapted an audio recording task from this platform, which was used to capture participants' recordings (https://app.gorilla.sc/openmaterials/417553). Our materials are available (https://app.gorilla.sc/openmaterials/740669).

#### Procedure

For the voice recording phase, participants completed a microphone check then recorded themselves repeatedly saying ‘hello’. Two tokens of ‘hello’ were extracted from each participant and used in the voice‐matching task. For the experimental testing phase, participants received a unique version of the experiment containing their own voice recordings alongside the two external voices. Six variations of the experiment randomly assigned the participant's own voice to either the Self, Friend or Stranger identity, with the two external voices counterbalanced in the remaining roles.

The experimental task procedure was identical to Experiment 1, except that participants completed an additional ‘own voice’ recognition test after completing the headphone check. This component involved two sequences each containing six voice samples, from which the participant had to correctly identify their own voice recordings before continuing to the Familiarization and Test phases. No participants were excluded for failing to recognize their own voice recordings at this stage.

### Results and discussion

As in Exp 1, reaction time and accuracy data are reported here, with sensitivity (D′) reported in Appendix S1. Means are summarized in Table 2, broken down by Trial Type (MATCH, MISMATCH), Own‐voice assignment (whether participant's own voice recording was assigned to the Self, Friend or Stranger identity) and Voice identity (whether the voice presented in the trial was that assigned to the Self, Friend or Stranger identity). To minimize model complexity, in both the RT and accuracy analysis we analysed Match and Mismatch trials separately, removing Trial Type as a fixed effect from our models. This pragmatic change from Experiment 1 is in line with previous convention when introducing additional factors, as their main effects and interactions are generally more observable in match trials (see Payne et al., 2021, Exp 3; Sui et al., 2012).

**TABLE 2:** Mean reaction time (in milliseconds) and proportionate accuracy, with 95% confidence intervals in parentheses.

Trial type	Own‐voice assignment	Voice identity	Reaction time	Accuracy
MATCH	SELF	Self^a^	494.3 [484.5, 504.1]	0.97 [0.96, 0.98]
		Friend	616.4 [601.8, 631.0]	0.89 [0.87, 0.91]
		Stranger	637.3 [622.1, 652.5]	0.83 [0.81, 0.85]
	FRIEND	Self	616.1 [602.3, 630.0]	0.88 [0.86, 0.90]
		Friend^a^	590.4 [578.0, 602.7]	0.92 [0.90, 0.94]
		Stranger	671.8 [655.8, 687.8]	0.86 [0.84, 0.88]
	STRANGER	Self	597.9 [583.0, 612.9]	0.85 [0.83, 0.87]
		Friend	635.8 [621.6, 649.9]	0.88 [0.86, 0.90]
		Stranger^a^	559.4 [548.5, 570.3]	0.92 [0.90, 0.94]
MISMATCH	SELF	Self^a^	596.9 [585.6, 608.2]	0.97 [0.96, 0.98]
		Friend	678.1 [664.4, 691.9]	0.84 [0.82, 0.86]
		Stranger	681.6 [667.2, 696.0]	0.83 [0.81, 0.85]
	FRIEND	Self	730.8 [716.2, 745.4]	0.86 [0.84, 0.88]
		Friend^a^	657.9 [645.3, 670.6]	0.94 [0.93, 0.95]
		Stranger	727.1 [712.5, 741.7]	0.86 [0.84, 0.88]
	STRANGER	Self	668.7 [655.1, 682.4]	0.83 [0.81, 0.85]
		Friend	685.1 [671.4, 698.8]	0.83 [0.81, 0.85]
		Stranger^a^	606.5 [595.4, 617.5]	0.94 [0.93, 0.95]

^a^ Indicates the condition to which participant's own voice was assigned.

#### Reaction times

Incorrect responses (11.7% of trials) and outliers in which reaction times were under 200 ms or over 1500 ms (a further 1% of trials) were removed. We separated the data by Trial Type (MATCH vs. MISMATCH) and conducted the following linear mixed effect model on both datasets separately:

$$\mathrm{rt}.\sim 1+\text{voice}\_\text{identity}\times \mathrm{own}\_\text{voice}\_\text{assignment}+\left(1\;|\;\text{Participant}\;\mathrm{ID}\right).$$

##### Match Trials

The full model output is available in Table S3. The model revealed a main effect of Voice identity (*χ* ^2^ (2) = 131.96, *p* < .001, *η* ^2^ = 0.02) with voices assigned to the Self identity eliciting faster responses than both Friend (*p* < .001, *d* = −0.42, 95% CI [−0.62, − 0.21]) and Stranger voices (*p* < .001, *d* = −0.47, 95% CI [−0.69, −0.25]), but no difference between Friend and Stranger voices (*p* = .052, *d* = −0.07, 95% CI [−0.28, 0.13]). There was no main effect of Own‐voice assignment (*χ* ^2^ (2) = 3.60, *p* = .165, *η* ^2^ = 0.04), but a significant Voice identity × Own‐voice assignment interaction (*χ* ^2^ (4) = 360.50, *p* < .001, *η* ^2^ = 0.04) indicated that the effect of Voice identity on RTs was dependent on the assignment of participants' own voice. Post‐hoc tests with Bonferroni adjustments for nine comparisons (adjusted significance threshold: *p* < .005) revealed the following patterns across Own‐voice assignment conditions:

##### Own voice assigned to SELF

Responses to the voice assigned to the Self identity were faster than both the Friend voice (*p* < .001, *d* = −1.13, 95% CI [−1.41, −0.84]) and Stranger voice (*p* < .001, *d* = −1.30, 95% CI [−1.69, −0.90]). The Friend–Stranger comparison did not reach the adjusted significance threshold (*p* = .009, *d* = −0.22, 95% CI [−0.45, 0.02]).

##### Own voice assigned to FRIEND

Responses to the voice assigned to the Friend identity (i.e. participants' own voice) were fastest, followed by responses to the Self voice (*p* = .004, *d* = 0.26, 95% CI [−0.11, 0.62]), both of which were faster than the Stranger voice (*p* < .001, *d* = −0.49, 95% CI [−0.79, −0.19] for Self–Stranger and *p* < .001, *d* = −0.70, 95% CI [−1.06, −0.33] for Friend–Stranger).

##### Own voice assigned to STRANGER

Responses to the voice assigned to the Stranger identity (i.e. participants' own voice) were fastest, followed by responses to the Self then Friend voices (*ps* < .001 for all three comparisons; *d* = −0.34, 95% CI [−0.61, −0.08] for Self–Friend; *d* = 0.36, 95% CI [0.07, 0.65] for Self–Stranger, and *d* = 0.75, 95% CI [0.40, 1.10] for Stranger–Friend).

**FIGURE 2:**
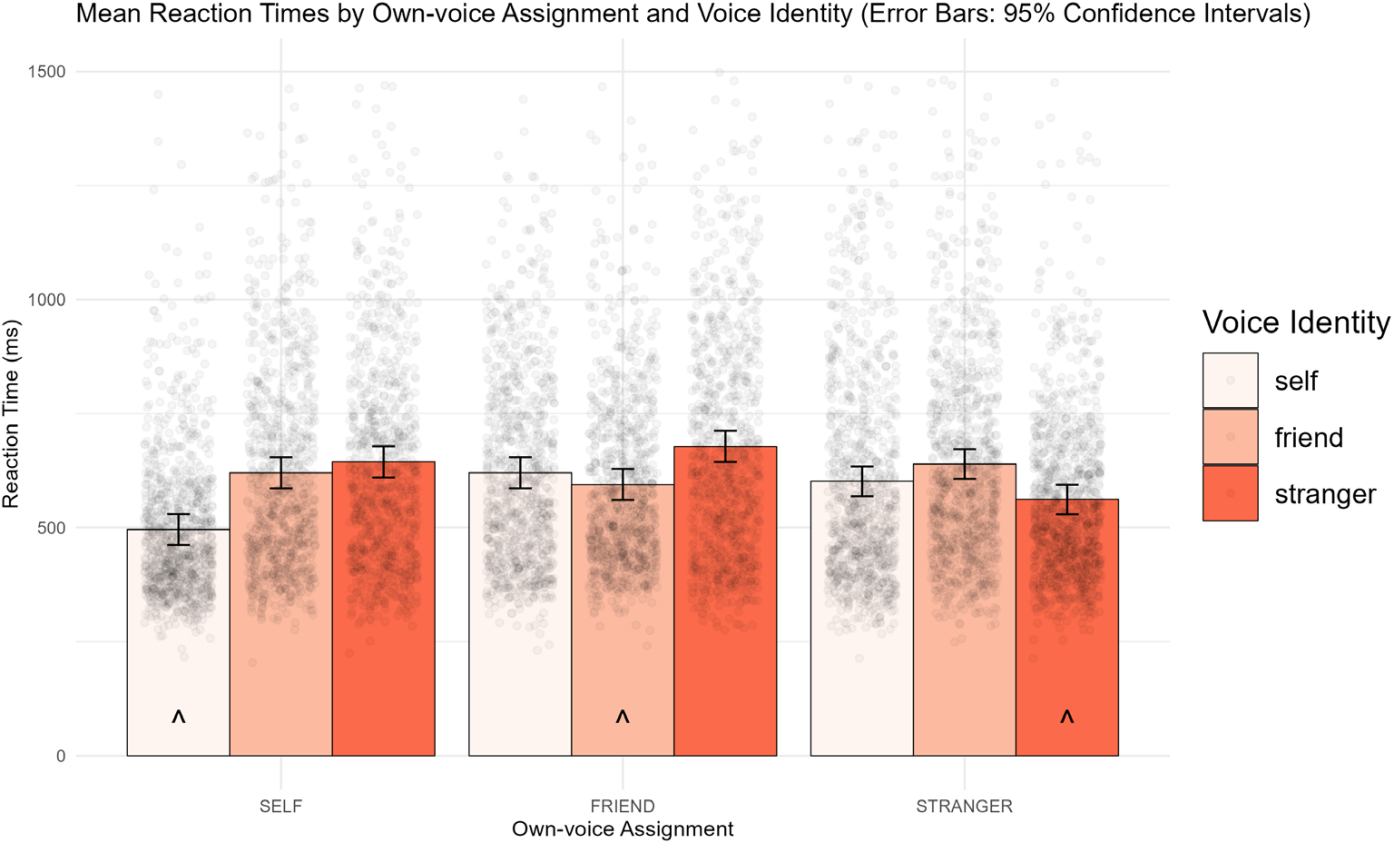
Mean RTs for each Voice identity across Own‐voice assignment conditions. Error bars indicate 95% confidence intervals and raw data points are overlaid. For each Own‐voice assignment condition, the ^ indicates the condition in which the participant's own voice appeared.

These patterns indicate that across all conditions, participants prioritized their own voice, regardless of whether it was assigned to Self, Friend or Stranger identities (see Figure 2). However, the voice associated with the Self identity was given higher priority than the other vocal cues when these were not presented in the participant's own voice.

In an additional pre‐registered analysis, we compared reaction time on trials featuring participants' own voices in the three separate Voice identity conditions. In other words, we took the three levels of Own‐voice assignment (SELF, FRIEND, STRANGER) and the relevant single level of Voice identity in which participant's own voice was presented, leading to a between‐subjects comparison of SELF/voice assigned to self‐identity, FRIEND/voice assigned to friend identity and STRANGER/voice assigned to stranger identity trials. This revealed that participants whose own voice was assigned to SELF were significantly faster when this cue was presented than participants whose own voice was assigned to FRIEND (*p* < .001, *d* = −1.05, 95% CI [−1.61, −0.50]) or STRANGER (*p* = .006, *d* = −0.83, 95% CI [−1.36, −0.31]). There was no significant reaction time difference between participants whose own voices were assigned to FRIEND and STRANGER (*p* = 0.174, *d* = 0.31, 95% CI = [−0.19, 0.82]). This demonstrates that although participants prioritized their own voice across conditions, this effect was boosted for those whose own voice was assigned as SELF.

Finally, we compared the categorization of the voice assigned to the Self identity across the three Own‐voice assignment conditions. For participants whose own voice was assigned to SELF, their own voice was also the voice assigned to the Self identity in the task. However, for those in the FRIEND and STRANGER own‐voice assignment conditions, an unknown external voice was the cue assigned to the Self identity. Participants' responses to the cue assigned to the Self identity were faster for those in the SELF own‐voice assignment condition compared to those whose own voices were assigned to the FRIEND (*p* < .001, *d* = −1.56, 95% CI [−2.15, −0.96]) and STRANGER conditions (*p* < .001 *d* = −1.11, 95% CI [−1.65, −0.56]). However, there was no significant difference between the responses to the Self identity cue in the FRIEND and STRANGER own voice assignment conditions (*p* = .438, *d* = 0.20, 95% CI [−0.31, 0.70]). This shows that when participant's own voice was assigned to the Self identity, this elicited a reaction time advantage relative to when external voices were assigned to this role.

Overall, Match trial data showed that the participants whose own voice was assigned to SELF received a significant boost in response time. Their responses on trials in which they heard their own voice was faster than that of participants whose own voices were assigned to FRIEND or STRANGER referents, and their responses to cues assigned to the self identity were faster than that of participants for whom as external voice was assigned to the self identity. Thus, the tendency to prioritize own‐voice cues across conditions was exacerbated in participants whose own voice was also the self‐identity cue.

##### Mismatch trials

MISMATCH RTs were also subject to the following model:

$$\mathrm{rt}.\sim 1+\text{voice}\_\text{identity}\times \mathrm{own}\_\text{voice}\_\text{assignment}+\left(1\;|\;\text{Participant}\;\mathrm{ID}\right).$$

The full model output is available in Table S4. The model showed a main effect of Own‐voice assignment (*χ* ^2^ (2) = 6.93, *p* = 0.031, *η* ^2^ = 0.07), although there were no significant differences between the groups after alpha adjustment (all *p*'s > .016). There was no main effect of Voice identity (*χ* ^2^ (2) = 2.41, *p* = .300, *η* ^2^ > .01) but a significant Own‐voice assignment × Voice identity interaction (*χ* ^2^ (4) = 323.75, *p* < .001, *η* ^2^ = 0.04), indicating that the effect of Voice identity on reaction times was dependent on the role to which the participant's own voice had been assigned. Post‐hoc tests with Bonferroni adjustments for nine comparisons (*p* < .005) revealed the following patterns across Own‐voice assignment conditions:

##### Own voice assigned to SELF

Responses to the voice assigned to the Self identity (i.e. participants' own voice) were significantly faster responses than responses to both the Friend (*p* < .001, *d* = −0.80, 95% CI [−1.07, −0.53]) and Stranger voices (*p* < .001, *d* = −0.83, 95% CI [−1.08, −0.59]). No significant difference was found between Friend and Stranger voices (*p* = .814, *d* = −0.02, 95% CI [−0.22, 0.18]).

##### Own voice assigned to FRIEND

Responses to the voice assigned to the Friend identity (i.e. participants' own voice) were faster than responses to both the Self (*p* < .001, *d* = 0.66, 95% CI [0.33, 0.99]) and Stranger voices (*p* < .001, *d* = −0.65, 95% CI [−1.03, −0.28]). There was no significant difference between Self and Stranger voices (*p* = .741, *d* = 0.04, 95% CI [−0.20, 0.27]).

##### Own voice assigned to STRANGER

The voice assigned to the Stranger identity (i.e. participants' own voice) elicited significantly faster responses than both Self (*p* < .001, *d* = 0.74, 95% CI [0.43, 1.04]) and Friend voices (*p* < .001, *d* = 0.83, 95% CI [0.53, 1.14]), while the comparison between Self and Friend voices was not significant (*p* = .167, *d* = −0.11, 95% CI [−0.40, 0.17]).

These patterns demonstrate that as in MATCH trials, the impact of Voice identity on reaction times in MISMATCH trials varied depending on the role assigned to the participants' own voice; no matter whether their own voice was assigned to SELF, FRIEND or STRANGER, participants were always faster at recognizing their *own* voice as being a mismatch with the on‐screen label, compared with the externally generated voices.

#### Accuracy

Accuracy data were submitted to the following general linear mixed effect model separately for both Match and Mismatch trials.

$$\text{Correct}\sim 1+\text{voice}\_\text{identity}\times \mathrm{own}\_\text{voice}\_\text{assignment}+\left(1\;|\;\text{Participant}\;\mathrm{ID}\right).$$

##### Match trials

The full model output is available in Table S5. The model revealed a main effect of Voice identity (*χ* ^2^ (2) = 26.32, *p* < .001), with voices assigned to the Self identity being more accurate than both Friend (*p* = .015, *d* = 0.01, 95% CI [−0.22, 0.24]) and Stranger voices (*p* < .001, *d* = 0.21, 95% CI [−0.07, 0.49]), and Friend voices being more accurate than Stranger voices (*p* = .004, *d* = 0.20, 95% CI [−0.04, 0.44]). As with the RT data, there was no main effect of Own‐voice assignment (*χ* ^2^ (2) = 2.46, *p* = .292), but a significant Voice identity × Own‐voice assignment interaction (*χ* ^2^ (4) = 177.07, *p* < .001). Post‐hoc tests with Bonferroni adjustments for nine comparisons (adjusted alpha – *p* < .005), revealed the following patterns:

##### Own voice assigned to SELF

Accuracy was higher for the voice assigned to the Self identity (i.e. participants' own voice) than both the Friend (*p* < .001, *d* = 0.89, 95% CI [0.41, 1.37]) and Stranger voices (p < .001, *d* = 1.24, 95% CI [0.62, 1.85]), with Friend also being more accurate than Stranger (*p* < .001, *d* = 0.40, 95% CI [0.00, 0.79]).

##### Own voice assigned to FRIEND

Accuracy for the voice assigned to the Friend identity (i.e. participants' own voice) was higher than both the Self (*p* = .001, *d* = −0.51, 95% CI [−0.99, −0.04]) and Stranger voices (*p* < .001, *d* = 0.55, 95% CI [0.07, 1.03]), with no differences between the Self and Stranger voices (*p* = .236, *d* = 0.15, 95% CI [−0.33, 0.64]).

##### Own voice assigned to STRANGER

Accuracy was higher for the voice assigned to the Stranger identity (i.e. participants' own voice) than both the Self (*p* < .001, *d* = −0.59, 95% CI [−0.92, −0.27]) and Friend voices (*p* = .002, *d* = −0.31, 95% CI [−0.70, 0.09]), with the difference between the Self and Friend voices non‐significant after alpha correction (*p* = 0.010, *d* = −0.27, CI [−0.56, 0.02]).

These patterns show that across all levels of Voice identity, participants were more accurate at recognizing that their *own* voice was a match with the on‐screen label, regardless of the referent with which it was associated.

##### Mismatch trials

The full model output is available in Table S6. The model revealed a main effect of Voice identity (*χ* ^2^ (2) = 13.03, *p* = .001) with voices assigned to the Self identity being more accurate than Friend (*p* < .001, *d* = 0.14, 95% CI [−0.08, 0.37]) and Stranger voices (*p* = .012, *d* = 0.04, 95% CI [−0.22, 0.30], and no difference between Friend and Stranger voices (*p* = .251, *d* = −0.11, 95% CI [−0.32, 0.10]). Again there was no main effect of Own‐voice assignment (*χ* ^2^ (2) = 1.30, *p* = .522) but a significant Own‐voice assignment × Voice identity interaction *χ* ^2^ (4) = 303.39, *p* < .001). Post‐hoc tests with Bonferroni adjustments for nine comparisons (adjusted alpha – *p* < .005) revealed the following patterns.

##### Own voice assigned to SELF

Accuracy was higher for the voice assigned to the Self identity (i.e. participants' own voice) than both Friend (*p* < .001, *d* = 1.19, 95%, CI [0.66, 1.73]) and Stranger voices (*p* < .001, *d* = 1.26, 95% CI [0.71, 1.82]), with no difference in accuracy between Friend and Stranger voices (*p* = .747, *d* = 0.04, 95% CI [−0.18, 0.27]).

##### Own voice assigned to FRIEND

Accuracy for the voice assigned to the Friend identity (i.e. participants' own voice) was higher than both the Self (*p* < .001, *d* = −0.88, 95% CI [−1.31, −0.45]) and Stranger voices (*p* < .001, *d* = 0.67, 95% CI [0.38, 0.97]), with no differences between the Self and Stranger voices (*p* = 0.933, *d* = −0.01, 95% CI [−0.31, 0.29]).

##### Own voice assigned to STRANGER

Accuracy was higher for the voice assigned to the Stranger identity (i.e. participants' own voice) than both the Self (*p* < .001, *d* = −0.68, 95% CI [−0.97, −0.39]) and Friend voices (*p* < .001, *d* = −1.03, 95% CI [−1.43, −0.63]), with no significant difference between the Self and Friend voices (*p* = .817, *d* = 0.01, 95% CI [−0.22, 0.24]).

As in the MATCH trials, accuracy data in the MISMATCH trials show a consistent pattern: across all levels of Own‐voice assignment, participants were more accurate at recognizing that their *own* voice was a mismatch with the on‐screen identity label, compared to the externally generated voices. Altogether, the findings from Exp 2 show that in both response time and accuracy, the bias for cues assigned to the self identity is boosted when that cue is participant's own voice. Further, in contrast to previous findings using visual cues (Cunningham et al., 2022), familiar own‐voice cues conferred more advantage on performance than the temporary self‐associations active during the task.

#### Exploratory analyses

##### Relationship between Acoustical Properties of Voice and Reaction Times

As an unregistered exploratory analysis, we investigated whether prioritization of the participants' own voice would be reduced if the external voice assigned to self had acoustic similarity to participants' own voice. We calculated the degree of acoustic similarity between the participants' own voice (only for those whose own voice was assigned to the FRIEND or STRANGER role) and the external voice that was assigned to the self identity. Using the concept of voice space first outlined by Baumann & Belin (2008), and recently utilized for self‐voices by Orepic et al. (2023), we used the phonTools R package (Barreda, 2023) to compute voice distance by extracting the means of the first five formants and the fundamental frequency (F0) from both the participants' and external speakers' audio files. We then used the following equations to compute the coordinates for each voice: *x* = log(F0); *y* = log(F5 – F4), before transforming them into z‐scores. To calculate voice distance, we used these coordinates to compute Euclidean distances by subtracting the (external) voice assigned to the Self identity from the participants' own real voice. The smaller the difference, the more acoustically similar the voices were.

We also calculated a percentage reaction time bias difference between the participants' real own voice and the external voice assigned to the Self identity. Inspired by the bias measures reported by Payne et al. (2021, Exp. 3), we took the mean reaction times for the Voice identity condition in which the participant's own voice appeared and subtracted this from the mean reaction times for the condition in which an external voice was assigned to the Self identity. We then divided this by the sum of the two conditions and multiplied this by 100 to achieve a percentage score. Higher percentage bias scores indicate a greater difference between reaction times for the (external) self‐associated voice, compared to the real (own) voice.

**FIGURE 3:**
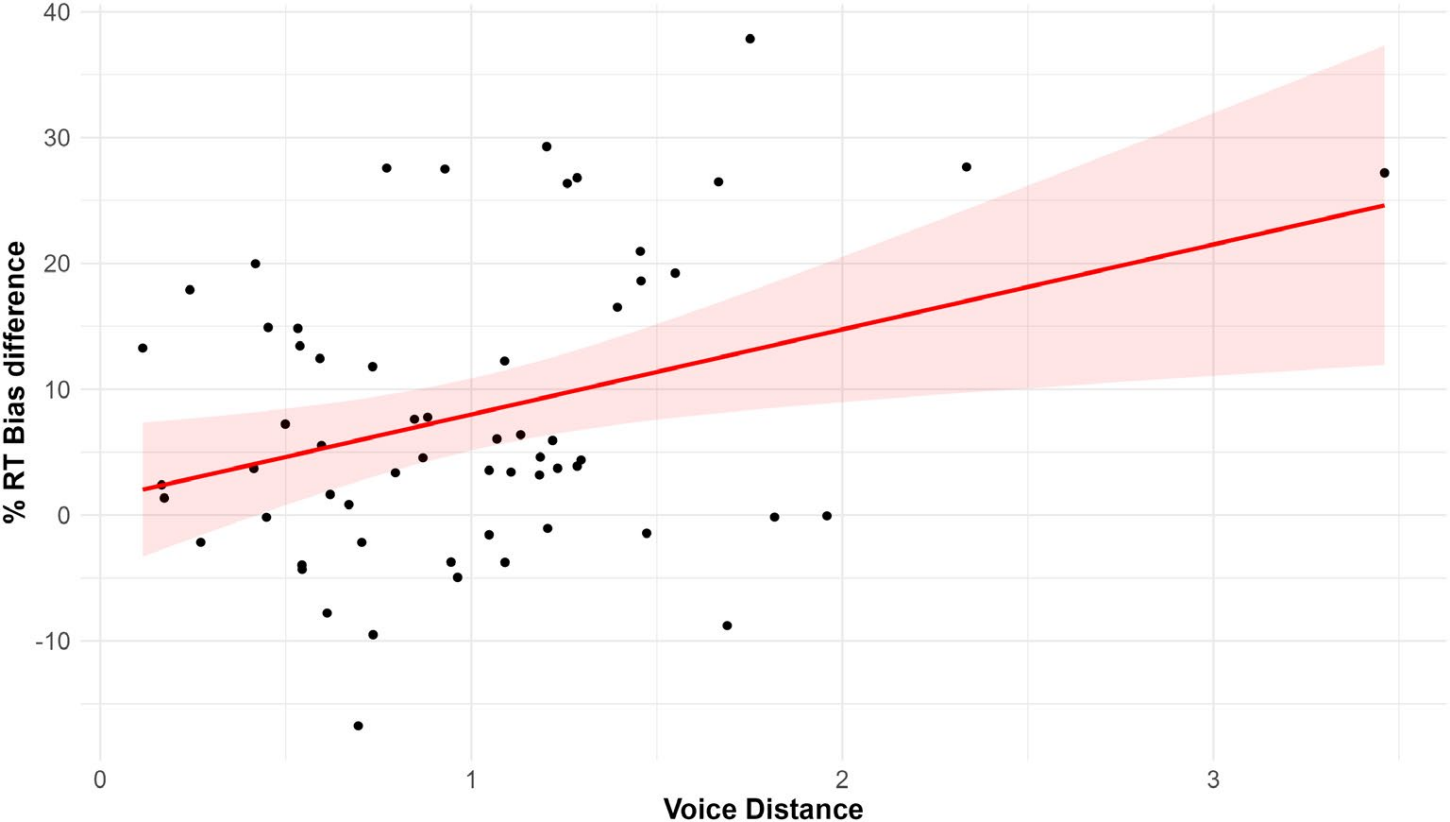
Scatter plot illustrating the relationship between Voice Space Distance and % Bias Difference for Reaction Times. Each point represents an individual participant's data with the red line depicting the best‐fit linear regression and the shaded area representing the 95% confidence interval.

We calculated a Pearson's correlation between voice distance and percent bias difference, which revealed a significant positive relationship (*r*(58) = .33, 95% CI [.09, .54], *p* = 009). In other words, participants have a reduced bias for their own voice when the external voice assigned to the Self identity is more similar to their own (see: Figure 3). We also computed this bias for accuracy data, but this did not show a significant relationship with voice distance (*r*(58) = −.07, 95% CI [−.32, .19], *p* = .613).

## GENERAL DISCUSSION

The current study demonstrates that participants have robust attentional biases to both voices assigned to the self identity and their own voice, with their own voice eliciting the greatest bias. In Experiment 1, we replicated Payne et al.'s (2021) finding that assigning an external voice to an on‐screen self identity label elicits faster and more accurate responses than assigning a voice to a friend or stranger identity label. This demonstrates that self‐prioritization effects in cognition are not limited to visual perception cues (Sui et al., 2012). Adapting Payne et al.'s paradigm to address a novel question, in Experiment 2 we demonstrated that participants prioritize their own voice over external voices, regardless of whether their own voice was assigned to self, friend or stranger. When the voiced assigned to the self identity was also the participants' own voice, this evoked the fastest responses. These findings have important theoretical and practical implications.

While own‐voice cues and self‐labelled voice cues were both associated with a processing advantage, there was clear evidence that participants' own voices elicited greater self‐bias. Specifically, Experiment 2 showed that own‐voice cues assigned to non‐self (i.e. friend and stranger) identities consistently elicited faster and more accurate responses than external voices assigned to the self identity label (although upcoming work suggests this may not always be the case; see Payne, 2022). Our finding suggests that a participant's own voice may be more effectively bound to the associated label than external voices, regardless of whether this label is assigned to self, friend or stranger. Sui et al. (2015) argue that self‐cues create an ‘integrative hub’, whereby active information is more likely to be integrated into bound representations, both in short‐term representations (e.g. visual features in perception) and in longer term memory (e.g. episodic recollection). The current findings add further weight to this argument, extending the findings from visual modality to verbal processing.

Notwithstanding the clear advantage for own‐voice trials over those with the voice assigned to the self identity, there was an additional boost to response times when own voices were assigned to Self. There are two potential explanations for this pattern. One is that vocal self‐prioritization effects can have an additive effect, with increased prioritization for cues that are more closely associated with self. This is consistent with previous research on self‐reference effects in memory and attention, which shows that evoking multiple levels of self‐association with a cue can increase self‐biases in cognition encoding (e.g. Cunningham et al., 2011, 2022). It may be supported by physiological responses to self‐cues, as own‐voice cues evoke an automatic affective response (Gur & Sackeim, 1979) that could operationalize the effects of self‐cues on in‐task alertness (Kaida & Abe, 2018). A second explanation is that when the self‐voice was assigned to a friend or stranger identity, this produced interference that reduced the prioritization effect relative to when the self‐voice was assigned to the self identity (see Devue et al., 2009; Röer et al., 2013). This account is supported by the finding that the prioritization of own‐voice cues was reduced when the external voice assigned to self was acoustically similar to a participant's own voice. It should be acknowledged that participants were aware that their voices had been recorded and their own‐voice recognition was tested before the experimental trials, potentially drawing attention to this stimulus. Separating these stages more substantially in time may be a useful design in future studies. However, the findings from the current paradigm clearly suggest that the cognitive effects of vocal self‐associations are significantly boosted when the associated cue is one's own voice.

The quality of participants' own voices that evoke this level of prioritization is an interesting theoretical question. One potential explanation is familiarity; we are highly familiar with the distinctive features of our own voice, allowing reliable recognition despite the acoustic qualities of a recorded version varying from a contemporaneous internal production (Holzman & Rousey, 1966; Kimura & Yotsumoto, 2018). Familiarity is an inherent feature of many self‐cues (e.g. own name, own face), a factor that has been argued to confound much research promoting a ‘self is special’ argument in terms of cognitive processing (see Gillihan & Farah, 2005; Golubickis & Macrae, 2023). Research suggests that when the self is contrasted with familiar others (e.g. mother, best friend), this can reduce the magnitude of self‐prioritization relative to distant or unfamiliar others (see Sui et al., 2012), suggesting that familiarity can be an important influence. This effect did not emerge consistently in the current study, although participants were not instructed to imagine a close friend in the ‘friend’ identity condition, perhaps reducing the extent to which this could elicit familiarity effects. While a fully factorial self × familiarity experimental design using participants' own voices is not possible in practice (i.e. participants' real voices cannot be assigned to an ‘unfamiliar’ condition), the effects of self and familiarity have been differentiated experimentally in previous research (e.g. Sui et al., 2014), suggesting these biasing mechanisms can operate independently of one another.

The prioritization of participants' own‐voices has important implications, including both clinical and practical applications. For example, the ability to differentiate self‐voices from vocalizations produced by others is associated with some neurological and psychiatric conditions (e.g. ‘hearing voices’ through auditory‐verbal hallucinations; see Iannotti et al., 2022; Orepic et al., 2023). Understanding the extent to which own‐voice cues elicit a cognitive response even when disassociated from self in healthy populations could provide a useful first step for studying this process in patients who have difficulties differentiating their own voice from that of others. Regarding the practical application of own‐voice cues, while previous research suggested that externally generated voices associated with self can elicit prioritization effects (Payne et al., 2021), the current findings show clearly that such effects are stronger when the voice cue is one's own. Additionally, our exploratory analysis revealed that when the voice assigned to the self identity was from an external person, similarity between that external voice and the participant's own voice reduced the response time difference between them. This relationship emerged even though participants were hearing their own voice through air rather than bone conduction (as occurs naturally during speech production; Orepic et al., 2023), demonstrating that the auditory change associated with air conduction did not eliminate their ability to benefit cognitively from own‐voice characteristics. This finding suggests that the use of digital technology to duplicate individual's own voices could confer benefits over other external voices in terms of increasing their ability to replicate cognitive self‐cue functionality, for example to support patients who use assistive technology to replace their natural voice, or for producing engaging avatars in automated digital systems or in creative industries and gaming. Artificial intelligence technology can produce vocal output that duplicates characteristics of individuals' own voices, and the current study provides robust evidence that such efforts are likely to provide measurably higher attentional bias and engagement than external voices, although this may be augmented by voices that are acoustically more similar to self. Future research should examine the extent to which this technology is associated with differential user experiences and behaviours in real‐world contexts.

In conclusion, the current study establishes that own‐voices are powerful self‐biasing tools, which elicit faster and more accurate processing than externally produced voice cues. While voices temporarily assigned to self evoke robust attentional biases, these effects are enhanced for own‐voice rather than external‐voice cues.

## AUTHOR CONTRIBUTIONS

**Neil W. Kirk:** Conceptualization; methodology; software; validation; funding acquisition; investigation; data curation; writing – review and editing; visualization; project administration; formal analysis. **Sheila J. Cunningham:** Conceptualization; methodology; writing – original draft; project administration.

## Supporting information


Appendix S1

## Data Availability

The data that support the findings of this study are openly available in Open Science Framework at https://osf.io/hr96d/, reference number DOI 10.17605/OSF.IO/HR96D.

## Footnotes

^1^
This power analysis was based on a medium‐sized interaction effect of $\eta_{p}^{2}$ = 0.06. With our final sample size, we observed a reduced medium‐sized effect of $\eta_{p}^{2}$ = 0.04.
