# Supplementary material for: Listen to yourself! Prioritization of self‐associated and own voice cues
Source: Br J Psychol. 2024 Oct 3;116(1):131–48. doi: 10.1111/bjop.12741 (PMC11724686; doi:10.1111/bjop.12741)
Supplement: Supplementary file 1 — Appendix S1 [file BJOP-116-131-s001.docx]

**Supplemental Materials for:**

**Listen to yourself! Prioritization of self-associated and own voice cues**

**Supplement 1: Additional details for Experiment 1**

**1 a) Participant Recruitment and Power Analysis**

As this was a replication study, we based our sample size and power analysis on the criteria reported in Experiment 1 of Payne et al. (2021) (n = 35). Participants were recruited through Prolific (www.prolific.ac) and were paid £3 GBP for completing this study.

**1 b) Data Processing**

The Gorilla Experiment Builder platform produces an output spreadsheet for every individual task (or questionnaire) component in its experiment tree. For Experiment 1 this means that participants’ experimental data are stored across six different downloadable task spreadsheets which need to be combined for data analysis. Data processing scripts, which combine these files and reduce the data to key variables for analysis are available from: DOI 10.17605/OSF.IO/HR96D.

**1 c) Additional Analyses**

*Sensitivity Analysis*: We pre-registered a sensitivity analysis in which correct MATCH trials were computed as “hits” and incorrect MISMATCH trials as “false alarms”. Hit rates and False Alarm rates were used to compute the signal detection d’ statistic for individual participant across each level of voice identity. We adjusted for cases involving 100% hits or 0% false alarms using the formula outlined by Stanislaw and Todorov (1999). As data from both MATCH and MISMATCH trials are used in this calculation, this factor is no longer used in our analysis. Sensitivity as measured by the d’ statistic was submitted to the following Linear Mixed Effect Model:

d_prime (sensitivity) ~ 1 + voice_identity (1 | Participant ID)

The full model output for sensitivity is available in Supplemental Table 7. The model revealed a significant main effect of voice identity (χ2 (2) = 6.77, p = .034). Post-hoc tests with a Bonferroni adjustment for 3 comparisons revealed a significant difference between sensitivity scores for the voices assigned to Self (mean = 3.2, 95% CI [3.0, 3.5] and Stranger (mean = 2.8, 95% CI [2.5, 3.2]) conditions (p = 0.14, *d* = 0.43, CI [0.09, 0.78]). Sensitivity for the Friend-associated voice (mean = 2.9, 95% CI [2.6, 3.3]) did not differ from either the Self (p = 0.06, *d* = 0.33 [-0.03, 0.70]) or Stranger voices (p = 0.59, *d* = 0.09, CI [-0.22, 0.41]).

**Supplement 2: Additional details for Experiment 2**

**2 a) Participant Recruitment Criteria and Power Analysis**

For this novel manipulation which included a between-subjects factor, we conducted a power analysis using MorePower (Campbell & Thomson, 2012) using the following criteria:

- Analysis - ANOVA
- Design Factors - RM = 3, IM = 3
- Effect of Interest - RM = 3, IM = 3
- Alpha (2 sides) = 0.05
- Sample = 126
- Effect Size - eta2 = .06

This power analysis indicated a medium-sized interaction effect between Own Voice Assignment and Voice Identity of η2 = .06 and sample size of 126 would achieve power of .9. With our final sample size (n = 90), we observed a reduced medium-sized effect of η2 = .04.

Participants were recruited through Prolific (www.prolific.ac) with eligibility based on the following self-reported criteria:

- Age 18-40
- Sex: Male
- Gender Identity: Male
- Nationality: English
- UK Area of Birth: East of England (East Anglia, Bedfordshire and Hertfordshire, Essex), South East, England (Berkshire, Buckinghamshire, and Oxfordshire, Surrey, Sussex, Kent, Hampshire and Isle of Wight)
- Current UK Area of Residence: East of England (East Anglia, Bedfordshire and Hertfordshire, Essex), London, England , South East, England (Berkshire, Buckinghamshire, and Oxfordshire, Surrey, Sussex, Kent, Hampshire and Isle of Wight), South West, England (Gloucestershire, Wiltshire and Bristol/Bath area, Dorset and Somerset, Cornwall and Isles of Scilly, Devon)
- English Speaking Monolingual: "I only know English"
- Hearing Difficulties: No
- Prolific Approval Rate: 99-100.

Participants were paid £0.90 GBP for completing Phase 1, and £3 GBP for completing Phase 2.

**2 b) Data Processing**

The Gorilla Experiment Builder platform produces an output spreadsheet for every individual task (or questionnaire) component in its experiment tree. As each participant in Experiment 2 completed a unique version of the task, this corresponds to 90 different task output spreadsheets that need to be combined. Data processing scripts, which combine these files and reduce the data to key variables for analysis are available for from: DOI 10.17605/OSF.IO/HR96D.

**2 c) Additional Methodology Details**

To ensure the rigor and replicability of our research, we have included more comprehensive methodological details in this Supplemental Materials section.

**Phase 2 (Main Task):** Participants received a unique version of the experiment containing their voice recordings alongside the two external voices. Six variations of the experiment were used, which assigned the participants’ own voice to either the Self, Friend or Stranger role, with the two external voices counterbalanced in the remaining roles as follows:

1. **Self - [Participant]**; Friend - M5; Stranger - M10
2. **Self - [Participant]**; Friend - M10; Stranger - M5
3. Self - M5; **Friend - [Participant]**; Stranger - M10
4. Self - M10; **Friend - [Participant]**; Stranger - M5
5. Self - M5; Friend - M10; **Stranger - [Participant]**
6. Self - M10; Friend - M5; **Stranger - [Participant]**

We created an R Markdown Script (DOI 10.17605/OSF.IO/HR96D) which organised the participants by an ID number (“Participant Private ID”) assigned to them by the Gorilla Experiment Builder platform in Phase 1. Gorilla assigns Participant Private ID numbers sequentially across all studies hosted on its platform, which means the Gorilla ID in our study reflects the order in which participants engaged with our research. Our script then looped through this pre-determined order, assigning participants to one of the six templates and incorporated their unique filenames into their personalised version of the task spreadsheet.

We set up the experiment tree so that six participants could be hosted within a single version of the experiment at a time, which branched them into their individual versions based on their unique usernames on Prolific. This meant that we did not have to host an individual experiment on the Prolific system for every single participant.

The rest of the experimental procedure was identical to Experiment 1, except participants completed an additional “own voice” recognition test after completing the headphone check. This component involved two sequences each containing six voice samples, from which the participant had to correctly identify their own voice recordings before continuing to the Familiarization and Test phases.

**2 d) Additional Analyses**

MATCH trials: Additional Pre-registered Reaction Time Analyses

*Comparison of participants’ own voice conditions:* In an additional pre-registered analysis, we compared reaction time on trials featuring participants’ own voices in the three separate Voice identity conditions. In other words, we took the three levels of Own-voice assignment (SELF, FRIEND, STRANGER) and the relevant single level of Voice identity in which participant’s own voice was presented, leading to a between-subjects comparison of SELF/self-associated, FRIEND/friend-associated, and STRANGER/stranger-associated trials.

This revealed that participants whose own voice was assigned to SELF were significantly faster when this cue was presented than participants whose own voice was assigned to FRIEND (p < .001, *d* = -1.05, 95% CI [-1.61, -0.50]) or STRANGER (p = .006, *d* = -0.83, 95% CI [-1.36, -0.31]). There was no significant reaction time difference between participants whose own voices were assigned to FRIEND and STRANGER (p = 0.174, *d* = 0.31, CI = [-0.19, 0.82]). This demonstrates that although participants prioritised their own voice across conditions, this effect was boosted for those whose own voice was assigned as SELF.

*Sensitivity Analysis:* For Experiment 2 we also pre-registered a sensitivity analysis in which correct MATCH trials were computed as “hits” and incorrect MISMATCH trials as “false alarms”. Hit rates and False Alarm rates were used to compute the signal detection d’ statistic for individual participant across each level of voice identity. We adjusted for cases involving 100% hits or 0% false alarms using the formula outlined by Stanislaw and Todorov (1999). As data from both MATCH and MISMATCH trials are used in this calculation, this factor is no longer used in our analysis.

Sensitivity as measured by the d’ statistic was submitted to the following Linear Mixed Effect Model:

d_prime (sensitivity) ~ 1 + voice_identity * own_voice_assignment (1 | Participant ID)

The full model output for sensitivity is available in Supplemental Table 8. The model revealed no significant main effects of voice identity (χ2 (2) = 5.85, p = .054) or own voice assignment (χ2 (2) = 0.90, p = .636) on sensitivity scores. However, there was a significant interaction between the two variables (χ2 (2) = 1.55, p = .462).

Post-hoc tests with a Bonferroni adjustment for 9 comparisons (adjusted alpha < .005), revealed that in each of the three categories of own voice assignment, participants had more sensitivity towards their own voice, compared to the other two external voices (all ps <.001), whereas there were no differences in sensitivity towards the two external voices across all categories of Participant Assignment (all ps > .09). See Supplemental Table 9 for a full list of p values and effect sizes.

**Appendices:**

Table S1: Results of the linear mixed-effects model using in Experiment 1 estimating reaction time (RT) based on voice identity (reference level = self) and trial type (reference level = Match). The table provides the estimate, standard error, t-statistic, degrees of freedom (df), and p-value for each fixed effect and interaction term.

| Model: rt ~ 1 + voice_identity * trialtype + (1 \| Participant ID) | | | | | |
| --- | --- | --- | --- | --- | --- |
| term | estimate | std.error | statistic | df | p.value |
| (Intercept) | 654.94 | 17.47 | 37.49 | 35.00 | < .001 |
| Voice identity friend | -22.43 | 3.39 | -6.61 | 6758.26 | < .001 |
| Voice identity stranger | 11.55 | 3.42 | 3.37 | 6758.30 | 0.001 |
| Trial Type Mismatch | -33.44 | 2.42 | -13.84 | 6758.38 | < .001 |
| Voice identity friend × Trial Type Mismatch | -18.63 | 3.39 | -5.49 | 6758.12 | < .001 |
| Voice identity stranger × Trial Type Mismatch | 6.88 | 3.42 | 2.01 | 6758.13 | 0.044 |

Table S2: Results of the General linear mixed-effects model used in Experiment 1 estimating accuracy based on voice identity (reference level = self) and trial type (reference level = Match). The table provides the estimate, standard error, t-statistic, and p-value for each fixed effect and interaction term.

| Model: Correct (Accuracy) ~ 1 + voice_identity * accent_match + (1 \| Participant ID) | | | | |
| --- | --- | --- | --- | --- |
| term | estimate | std.error | statistic | p value |
| (Intercept) | 2.56 | 0.14 | 18.04 | < .001 |
| Voice identity friend | 0.33 | 0.06 | 5.18 | < .001 |
| Voice identity stranger | -0.11 | 0.06 | -1.82 | 0.069 |
| Trial Type Mismatch | 0.22 | 0.04 | 5.22 | < .001 |
| Voice identity friend × Trial Type Mismatch | 0.07 | 0.06 | 1.11 | 0.267 |
| Voice identity Stranger × Trial Type Mismatch | 0.02 | 0.06 | 0.39 | 0.694 |

Table S3: Results of the linear mixed-effects model for the MATCH trial type in Experiment 2, estimating reaction time (RT) based on own voice assignment (reference level = SELF) and voice identity (reference level = self). The table provides the estimate, standard error, t-statistic, degrees of freedom (df), and p-value for each fixed effect and interaction term.

| Model: rt ~ 1 + voice_identity * own_voice_assignment + (1 \| Participant ID) | | | | | | |
| --- | --- | --- | --- | --- | --- | --- |
| Term | Estimate | | Std. Error | Statistic | df | P.Value |
| (Intercept) | 606.16 | | 9.47 | 90.13 | 63.99 | < .001 |
| Voice identity friend | | -33.67 | 2.96 | 8460.15 | -11.39 | < .001 |
| Voice identity stranger | 21.84 | | 2.98 | 8461.75 | 7.32 | < .001 |
| Own Voice Assignment FRIEND | -19.45 | | 13.50 | 90.09 | -1.44 | 0.15 |
| Own Voice Assignment STRANGER | 24.75 | | 13.50 | 90.11 | 1.83 | 0.07 |
| Voice identity friend × Own Voice Assignment FRIEND | -57.26 | | 4.17 | 8460.30 | -13.74 | < .001 |
| Voice identity stranger × Own Voice Assignment friend | 35.61 | | 4.27 | 8461.85 | 8.33 | < .001 |
| Voice identity friend × Own Voice Assignment STRANGER | 22.98 | | 4.23 | 8460.20 | 5.44 | < .001 |
| Voice identity stranger × Own Voice Assignment STRANGER | 25.30 | | 4.26 | 8461.73 | 5.94 | < .001 |

Table S4: Results of the linear mixed-effects model for the MISMATCH trial type in Experiment 2, estimating reaction time (RT) based on own voice assignment (reference level = SELF) and voice identity (reference level = self). The table provides the estimate, standard error, t-statistic, degrees of freedom (df), and p-value for each fixed effect and interaction term.

| Model: rt ~ 1 + voice_identity * own_voice_assignment + (1 \| Participant ID) | | | | | |
| --- | --- | --- | --- | --- | --- |
| Term | Estimate | Std. Error | Statistic | df | P.Value |
| (Intercept) | 674.82 | 9.26 | 72.90 | 89.88 | < .001 |
| Voice identity friend | -4.19 | 2.90 | -1.44 | 8345.55 | 0.149 |
| Voice identity stranger | 0.65 | 2.91 | 0.22 | 8345.10 | 0.824 |
| Own Voice Assignment FRIEND | -19.37 | 13.20 | -1.47 | 89.86 | 0.146 |
| Own Voice Assignment STRANGER | 35.39 | 13.19 | 2.68 | 89.82 | 0.009 |
| Voice identity friend × Own Voice Assignment FRIEND | -52.83 | 4.09 | -12.93 | 8345.98 | < .001 |
| Voice identity stranger × Own Voice Assignment FRIEND | 28.93 | 4.17 | 6.94 | 8344.92 | < .001 |
| Voice identity friend × Own Voice Assignment STRANGER | 29.60 | 4.15 | 7.14 | 8344.98 | < .001 |
| Voice identity stranger × Own Voice Assignment STRANGER | 21.82 | 4.14 | 5.27 | 8344.71 | < .001 |

Table S5: Results of the General linear mixed-effects model for the Match trial type in Experiment 2, estimating Accuracy based on own voice assignment (reference level = SELF) and voice identity (reference level = self). The table provides the estimate, standard error, t-statistic, and p-value for each fixed effect and interaction term.

| Model: Correct (Accuracy) ~ 1 + voice_identity * own_voice_assignment + (1 \| Participant ID) | | | | | | |
| --- | --- | --- | --- | --- | --- | --- |
| Term | Estimate | Std. Error | Statistic | P.Value |  |  |
| (Intercept) | 2.432 | 0.097 | 25.036 | < .001 |  |  |
| Voice identity friend | 0.237 | 0.058 | 4.103 | < .001 |  |  |
| Voice identity stranger | -0.237 | 0.050 | -4.771 | < .001 |  |  |
| Own Voice Assignment FRIEND | 0.220 | 0.139 | 1.581 | 0.114 |  |  |
| Own Voice Assignment STRANGER | -0.124 | 0.136 | -0.909 | 0.363 |  |  |
| Voice identity friend × Own Voice Assignment FRIEND | 0.971 | 0.096 | 10.097 | < .001 |  |  |
| Voice identity stranger × Own Voice Assignment FRIEND | -0.606 | 0.073 | -8.354 | < .001 |  |  |
| Voice identity friend × Own Voice Assignment STRANGER | -0.360 | 0.075 | -4.817 | < .001 |  |  |
| Voice identity stranger × Own Voice Assignment STRANGER | -0.045 | 0.068 | -0.660 | 0.509 |  |  |

Table S6: Results of the General linear mixed-effects model for the Mismatch trial type in Experiment 2, estimating Accuracy based on own voice assignment (reference level = SELF) and voice identity (reference level = self).The table provides the estimate, standard error, t-statistic, and p-value for each fixed effect and interaction term.

| Model: Correct (Accuracy) ~ 1 + voice_identity * own_voice_assignment + (1 \| Participant ID) | | | | | | |
| --- | --- | --- | --- | --- | --- | --- |
| Term | Estimate | Std. Error | Statistic | P.Value |  |  |
| (Intercept) | 2.43 | 0.11 | 21.95 | < .001 |  |  |
| Voice identity friend | 0.19 | 0.06 | 3.36 | 0.001 |  |  |
| Voice identity stranger | -0.05 | 0.05 | -0.93 | 0.353 |  |  |
| Own Voice Assignment FRIEND | 0.15 | 0.16 | 0.97 | 0.333 |  |  |
| Own Voice Assignment STRANGER | 0.00 | 0.16 | 0.00 | 0.997 |  |  |
| Voice identity friend × Own Voice Assignment FRIEND | 1.14 | 0.10 | 11.94 | < .001 |  |  |
| Voice identity stranger × Own Voice Assignment FRIEND | -0.64 | 0.07 | -8.73 | < .001 |  |  |
| Voice identity friend × Own Voice Assignment STRANGER | -0.53 | 0.08 | -7.08 | < .001 |  |  |
| Voice identity stranger × Own Voice Assignment STRANGER | -0.28 | 0.07 | -3.94 | < .001 |  |  |

Table S7: Summary of Linear Mixed-Effects Model Predicting Sensitivity (d-prime) as a function of Voice identity (reference level = self) from Experiment 1. The model includes random intercepts for Participant ID.

| Model: Sensitivity (d prime) ~ 1 + voice_identity + (1 \| Participant ID) | | | | |
| --- | --- | --- | --- | --- |
| term | estimate | std.error | statistic | p value |
| (Intercept) | 2.98 | 0.13 | 23.56 | < .001 |
| Voice identity friend | 0.24 | 0.09 | 2.60 | 0.011 |
| Voice identity stranger | -0.07 | 0.09 | -0.77 | 0.446 |

Table S8: Summary of Linear Mixed-Effects Model Predicting Sensitivity (d-prime) based on own voice assignment (reference level = SELF) and voice identity (reference level = self). Voice identity and Own Voice Assignment from Experiment 2. The model includes random intercepts for Participant ID.

| Model: sensitivity (d prime) ~ 1 + voice_identity * own_voice_assignment + (1 \| Participant ID) | | | | | |
| --- | --- | --- | --- | --- | --- |
| Term | Estimate | Std. Error | Statistic | df | P.Value |
| (Intercept) | 2.79 | 0.09 | 31.03 | 90.00 | < .001 |
| Voice identity friend | 0.12 | 0.05 | 2.18 | 180.00 | 0.031 |
| Voice identity stranger | -0.11 | 0.05 | -2.04 | 180.00 | 0.043 |
| Own Voice Assignment FRIEND | 0.10 | 0.13 | 0.81 | 90.00 | 0.421 |
| Own Voice Assignment STRANGER | 0.00 | 0.13 | 0.01 | 90.00 | 0.994 |
| Voice identity friend × Own Voice Assignment FRIEND | 0.92 | 0.08 | 12.03 | 180.00 | < .001 |
| Voice identity stranger × Own Voice Assignment FRIEND | -0.55 | 0.08 | -7.20 | 180.00 | < .001 |
| Voice identity friend × Own Voice Assignment STRANGER | -0.42 | 0.08 | -5.50 | 180.00 | < .001 |
| Voice identity stranger × Own Voice Assignment STRANGER | -0.13 | 0.08 | -1.68 | 180.00 | 0.09 |

Table S9: Pairwise comparisons of Sensitivity scores (d’) for different Own Voice Assignment and voice identities from Experiment 2. P values and Cohen’s *d* effect sizes (with 95% Confidence Intervals in parentheses) p-values are provided for each contrast within the three groups of Own Voice Assignment (their own voice in the role of SELF, FRIEND, or STRANGER).

| Own Voice Assignment | contrast | pvalue | Effect Size [& 95% CI] |
| --- | --- | --- | --- |
| SELF | self - stranger | < .001 | 1.46 [0.89, 2.02] |
|  | self - friend | < .001 | 1.7 [1.07, 2.32] |
|  | stranger - friend | 0.091 | 0.25 [0.02, 0.47] |
| FRIEND | self - stranger | 0.691 | -0.88 [-1.28, -0.48] |
|  | self - friend | < .001 | -0.06 [-0.41, 0.29] |
|  | stranger - friend | < .001 | 0.69 [0.35, 1.03] |
| STRANGER | self - stranger | < .001 | -0.17 [-0.38, 0.05] |
|  | self - friend | 0.221 | -0.86 [-1.15, -0.57] |
|  | stranger - friend | < .001 | -0.74 [-1.1, -0.37] |
